# The academic and health policy conference on correctional health: evaluation of its academic and scientific impact

**DOI:** 10.1186/s40352-015-0029-z

**Published:** 2015-10-05

**Authors:** Judith A. Savageau, Warren J. Ferguson, Laura Sefton

**Affiliations:** University of Massachusetts Medical School, Worcester, MA USA

## Abstract

**Background:**

There is limited research and research dissemination on the care of detained persons, often due to barriers to conducting research in correctional settings. Additionally, while concerns exist about the quality of care delivered to inmates, only a small number of academic health science centers provide health care services behind bars. To strengthen the field of academic criminal justice health (ACJH), the Academic and Health Policy Conference on Correctional Health (AHPCCH) was launched in 2007. Objective: To assess the merits of the conference as a stimulus to advance the field of ACJH.

**Methods:**

Two hundred ninety-one individuals were identified who had presented at the AHPCCH and/or had received a conference attendance scholarship between 2011 and 2013. A web-based survey assessed: networking opportunities; motivation to disseminate or continue in this field; scholarly outputs; clinical practice changes; clinical guidelines development; curriculum/training opportunities; and a climate assessment at participant’s home institution in support of their work.

**Results:**

With a 56 % response rate, the majority felt that the conference: provided encouragement and confidence to continue their work; validated their identity as a contributor in the field; and provided valuable feedback on their work. 86 % reported that the conference provided numerous networking opportunities. Most respondents reported that the conference provided new ideas for research and/or academic efforts and 62 % reported motivation to expand their scholarly work. Most also indicated that their choice to work in criminal justice health was respected at their home institution, with 64 % identifying collaborators with similar content interest/expertise and 66 % reporting opportunities to advance available as a result of their work. However, 70 % do not receive institutional funding during periods when their own extramural funding is low and 59 % were not part of an ACJH research core.

**Conclusions:**

The majority of presenters and scholars felt that the conference fulfilled professional development opportunities needed in the field. Moreover, the conference generated new ideas for research and/or academic efforts. Thus, the AHPCCH is a valuable opportunity for researchers, policymakers and clinicians to network, share and improve upon their work, generate research ideas and, ultimately, validate criminal justice health as an academic field of study.

## Background

The number of people under correctional supervision has grown to over three percent of the adult population in the United States (Clear et al. 2012). On any given day, it is estimated that one in every 108 adults are in prison or jail (Glaze and Herberman 2013) and this risk substantially increases for people of color and ethnic minorities (Office of National Drug Control Policy 2009). Drug-defined and drug-related offenses as well as offenses arising from a drug-using lifestyle are estimated to be 85 % of the 2.3 million people in prison who are substance-involved (The National Center on Addiction and Substance Abuse at Columbia University 2010). Moreover, with approximately 14.5 % of incarcerated people having a serious mental illness (Lamb and Weinberger 1998), co-occurring illness is common. Thirty to forty percent of detainees have a chronic medical condition including infections spread through injection drug use (Wang et al. 2014).

Although availability of health care while incarcerated has been deemed to be a constitutional right (Estelle v. Gamble 1976; Bell v. Wolfish^1979^) and despite the high prevalence of incarceration, there is limited research and research dissemination on evidence-based practices in the care of detained persons (Kendig 2004). Some of the limited research results from the lack of federal funding for research related to healthcare of incarcerated individuals (Ahalt et al. 2015). In addition to limited funding opportunities, barriers to conducting research in a secure environment are multiple and include stringent human subject protections (Thomas 2010), the security mission, and concerns about adverse study results which could result in litigious behavior.

Beyond research, historically there has been much criticism about the quality of care delivered in penal systems, citing lack of systematic quality assurance and improvement systems as well as accreditation standards. The National Commission on Correctional Health Care (NCCHC) pioneered efforts to create such standards and both NCCHC and the American Correctional Association now have accreditation standards for health care taking place in jails and prisons. Yet, as recently as 15 years ago, prison systems have been accused of hiring incompetent physicians both in the United States and in the United Kingdom (Dabney and Vaughn 2000).

The University of Massachusetts Medical School has been involved in work with health care systems providing care for detained populations for 15 years, providing direct care to inmates in Massachusetts prisons and also administratively supporting care provided to inmates in the Federal Bureau of Prisons. Academic health centers, particularly those that are publicly-funded institutions, share a mission to treat disadvantaged populations, to train the next generation of clinicians, and to develop and disseminate new knowledge to reduce the burden of disease and improve the health of individuals and populations. Incarcerated populations have the most prevalent burden of disease and health disparities in the United States, even in comparison with inner-city populations. Yet, only a small number of states have contracted with academic health science centers to provide health care services behind bars despite the potential benefits for this model. Those institutions who have made this commitment benefit by improving their population management systems, providing excited learners with unique core competencies, expanding their research agendas, and increasing their revenue base (Trestman et al. 2015). Among these institutions, there have been calls for a stronger collaboration between academic medicine and correctional health systems (Kendig 2008). Despite such encouragement and an identified need articulated by the National Academy of Science (Travis et al. 2014), there has been limited extramural funding to conduct research in the field (Ahalt et al. 2015). Moreover, clinicians and research faculty concerned with criminal justice health anecdotally report marginalization by institutional and academic medicine. To address these problems and to strengthen the field of academic criminal justice health, the Academic and Health Policy Conference on Correctional Health (AHPCCH) was developed and launched in 2007.

Now in its eighth year, the AHPCCH is an interdisciplinary, peer-reviewed conference focused on health care, policy, research and training innovations in correctional health. With 200–250 participants annually, the diversity of institutions, states and countries has expanded along with the number of peer-reviewed presentations resulting from investigator-initiated research. In 2011, a scientific meeting grant was awarded for four years and the conference was redesigned, guided by the following aims: to encourages high quality, interdisciplinary research in correctional health; to engage an interdisciplinary group of participants to network and share experiences; to learn from each others’ successes and failures; to discuss ideas for interventions to improve outcomes for detainees; and to facilitate the development of interdisciplinary research teams to design research protocols, conduct research, and disseminate and adopt evidence-based clinical practices in correctional settings and post release that emerge from these activities. Additionally, with grant support, competitive scholarships to attend the conference for junior investigators and students conducting work and interested in the field have been offered. Publication of selected proceedings was facilitated periodically through an agreement with the Journal of Correctional Health Care and announced to conference presenters annually and via a Call for Papers commentary (Miles 2012).

The conference organizers (including one of the authors/WF) conducted a structured evaluation of the conference each year, centering on participant demographics, reasons for attending, quality of the presentations, and overall acceptability. Participants ranked networking and the conference content as the two greatest motivators for attending. Very positive feedback describing AHPCCH as an academic home, fostering new collaborations and idea generation, have been consistently received. However, evaluations have not formally assessed the true academic impact of the conference or on health care practices and policies behind the four walls.

Scientific meetings provide an opportunity to network with peers, which can lead to a generation of new research ideas and collaborations. Additionally, presenting current research at conferences allows presenters to disseminate findings to a large audience and receive valuable feedback from peers. This feedback can be considered when developing manuscripts, leading to stronger submissions and a better likelihood of publication (Wood and Morrison 2011; Scherer et al. 2007). Some studies have shown that clinical results presented at conferences have influenced changes in clinical practice (Kattachchi and Nguyen 2010).

Guided by these purposes for scientific meetings and informal feedback from researchers and clinicians in the field, our study addressed the following questions: Have participants in the field been inspired by and do they feel that their work has been validated at the conference? Has the conference facilitated idea generation and new interdisciplinary research? Have evidence-based clinical guidelines been translated into policy or practice? Surveying presenters provides an important opportunity to further assess the merits of the conference as a stimulus for collaborative, innovative and interdisciplinary research. Given the barriers to academic careers focused on criminal justice health, we were also interested in the value of the conference to scholars as they choose career paths. Developing a deeper understanding of their needs serves as a potential opportunity for future grant funding.

## Methods

### Study population and recruitment

In an effort to decrease potential recall bias by surveying participants for all eight years the Academic and Health Policy Conference on Correctional Health (AHPCCH) has been held, we used summative registration documents for the three most recent years: 2011–2013. The selection of the study time frame was also related to the re-design of the conference in 2011 upon receipt of an NIH-funded scientific meeting grant. We identified 291 individuals who had either presented at one of the three conferences and/or had been a recipient of a scholarship funding their conference attendance. We postulated that this subset of conference attendees (i.e., presenters and scholars) would be more likely to be committed to careers in academic criminal justice health. An email letter of introduction was sent to all potential respondents with a link to the survey instrument. At two-week intervals, two reminder emails were sent to survey non-responders along with a link to the survey. A final (fourth) email was sent to non-responding conference attendees clarifying the intent of the survey and differentiating it from the most recent 2014 AHPCCH evaluation survey which had been fielded shortly before our initial survey mailing. Recruitment took place between April and June of 2014.

### Survey design

A focus group was held to assist with the development of our survey questions. Three former AHPCCH presenters (from years previous to our study timeframe, but including individuals still engaged in correctional health) were selected based on their regional location. The focus group method was chosen to provide an environment for a collective generation of ideas and discussion about potential survey questions. Suggestions forthcoming on questions to be asked included the categories of motivation, feedback, validation, networking, collaboration, and scholarship. Detailed note-taking occurred rather than audio-recording and a verbatim transcription of the focus group’s discussions. As the number of focus group attendees was small, the qualitative data were analyzed using strategies outlined by Patton (2001) which transformed the notes into survey questions. Based on this analysis, our survey was drafted and subsequently reviewed for content by members of the governing body that oversees the conference, the Academic Consortium on Criminal Justice Health. Upon final editing, the survey was developed for implementation within SurveyMonkey – a web-based survey development and data gathering tool (SurveyMonkey, Inc. Palo Alto, CA). The survey received a final review by our focus group participants for flow and content, and revisions made as needed.

The survey included three sections: 1) a 26-item section designed specifically for individuals who had presented at any one of the three conferences between 2011 and 2013; 2) a 6-item section designed specifically for individuals who had received at least one scholarship to attend any of the three conferences during the study years; and 3) an 18-item section for both presenters and scholarship recipients. For all respondents, survey items included: demographic data, years of experience in academia and criminal justice heath, years of conference attendance, scholarly outputs as a result of attending the conference, and a number of questions assessing the climate of criminal justice health at the participant’s home institution (e.g., availability of collaborators, mentorship, opportunities for funding, opportunities for advancement, and respect offered regarding working in this field). Presenters were specifically asked about: feedback and validation received as a result of presenting at the conference; opportunities for networking; motivation to disseminate their work further and/or continue their work; scholarly outputs (e.g., publications, presentations, grants, and new collaborations); and other tangible outcomes (e.g., changes in clinical practice, development of new clinical policies or guidelines in criminal justice health, and development of new or expanded curriculum or training opportunities in criminal justice health). Finally, scholarship recipients were asked about: importance of receiving a scholarship; continued work in criminal justice health since attending the conference; and impact on recognition/visibility at their home institution.

### Data analysis

Survey data were analyzed using SPSS statistical software (IBM SPSS Statistics for Windows, Version 22.0. Armonk, NY; 2013). Frequency distributions and measures of central tendency were used to describe the study population and their responses to individual survey items. Bivariate statistical tests (chi-square tests and t-tests, depending on the categorical or continuous nature of the variables) were used to analyze respondent characteristics associated with select study questions. Responses to questions gathered through the use of five-point Likert scales were dichotomized. For example, when asked “To what extent did presenting at the AHPCCH…” “provided me with valuable feedback on my work”; “provided me with networking opportunities with funders”; or (among others) "motivated me to prepare a manuscript”, we collapsed responses of ‘Strongly disagree’/‘Disagree’/‘Neutral’ versus ‘Agree somewhat’/‘Strongly agree’ as well as ‘Not at all'  = ‘1’/ ‘2’/‘3’ versus ‘4’/‘A great deal’. While we intended to conduct bivariate analyses comparing responses from presenters and scholarship recipients where there was no overlap between the two groups, nearly all (37 of 40) scholarship recipients were also presenters; thus, this analysis was not conducted. A p-value ≤0.05 was used as the threshold to indicate statistical significance.

The University of Massachusetts Medical School Institutional Review Board (IRB) reviewed our study proposal and determined it to be ‘non-human subjects research’ as an evaluation of the AHPCCH conference. Thus, no further IRB review/oversight was needed throughout the tenure of the project.

## Results

Of the initially sampled 291 conference presenters and scholarship recipients, a total of 34 individuals were subsequently deleted from the respondent pool because of: 1) our contact information was not correct and they never received the survey; 2) they were initially registered as presenters at the conference but had not actually presented; or 3) they ultimately did not attend the conference. This led to a response rate of 54.5 % (140/257). Another 7 surveys were subsequently deleted as less than one-half of the survey was completed and attempts to reach the participant failed; thus, our completion rate was 56.0 % (140/250).

### Demographic characteristics

As shown in Table 1, our survey respondents were: nearly evenly split by gender; had a mean age of 47.2 (SD: 11.9); almost exclusively non-Hispanic; and three-quarters white. The disciplines in which these respondents currently worked ranged from a few participants in law, sociology, pharmacy, and quality assurance/improvement to the most prevalent categories being behavioral health (28.6 %), public health (31.4 %) and research (32.1 %). Respondents often had multiple roles in their current position: researcher/policy expert (57.7 %), clinician/educator (45.3 %), and administrator/executive leader (24.1 %). Respondents reported a wide range in terms of years in academia as well as in criminal justice health (academia: mean: 11.3; SD: 8.8; median: 9.5; range: 0-40 years; criminal justice health: mean: 11.4; SD: 8.6; median: 9.5; range: 1–41 years). Finally, just over one-half (54.1 %) of respondents only attended one AHPCCH; the remaining attended either two (33.1 %) or three (12.8 %) of the conferences within our study timeframe.

**Table 1:** Sociodemographic and academic characteristics of AHPCCH conference attendees; *N* = 140, 2014

	Study sample^a^ *N* (%)
Sociodemographic characteristics	
Gender	
Male	61 (45.9)
Female	72 (54.1)
Age group	
Under 30 years	7 (5.6)
30–39 years	30 (24.2)
40–49 years	32 (25.8)
50–59 years	33 (26.6)
60+ years	22 (17.7)
Range	25–73 years
Mean (SD)	47.2 (11.9)
Median	46.5
Race	
White	107 (81.7)
Non-White	24 (18.3)
Ethnicity	
Not Hispanic or Latino	125 (97.7)
Hispanic or Latino	3 (2.3)
Academic characteristics	
Discipline	
Behavioral health (e.g., psychology, social work)	40 (28.6)
Education	10 (7.1)
Health management	11 (7.9)
Infectious diseases	15 (10.7)
Law	2 (1.4)
Nursing	19 (13.6)
Pharmacy	1 (0.7)
Primary care	21 (15.0)
Psychiatry	8 (5.7)
Public health	44 (31.4)
Quality assurance/improvement	6 (4.3)
Research	45 (32.1)
Sociology	6 (4.3)
Other medical specialties (e.g., pediatrics, OB/Gyn)	9 (6.4)
Other non-medical specialties (e.g., IT, economics)	11 (7.9)
Current role at home institution	
Clinician	39 (27.9)
Educator	47 (33.6)
Researcher	76 (54.3)
Policy expert	15 (10.7)
Administrator	26 (18.6)
Executive leader	17 (12.1)
Other (e.g., consultant, student, funder)	15 (10.7)
Years of experience in academe	
Range	0–40 years
Mean (SD)	11.3 (8.8)
Median	9.5
Years of experience in criminal justice health	
Range	1–41 years
Mean (SD)	11.4 (8.6)
Median	9.5
Conference characteristics	
Presenters	137 (97.9)
Scholarship recipients^b^	40 (28.6)

^a^Study Sample: Some variables may not total to 140 because of sporadic missing data

^b^Of the 40 scholarship recipients, 37 (92.5 %) also were conference presenters

### Conference presenters

Nearly all (137 of 140) respondents presented at one of the AHPCCH. Table 2 details the many advantages they reported from presenting at the conference. For example, the vast majority felt that the conference: provided encouragement to continue their work (83.9 %), validated their identity as a contributor in the field of criminal justice health (73.0 %), provided valuable feedback on their work (71.3 %), improved their confidence (64.7 %), and provided constructive feedback to improve their work (61.8 %). Equally positive responses were reported regarding networking opportunities; i.e., 86.0 % reported that the conference provided networking opportunities with individuals with similar interests while 75.7 % reported the conference provided networking opportunities with senior faculty. Reports on motivation were less positive overall than feedback, validation and networking. Most presenters (78.8 %) reported that the conference provided new ideas for research and/or academic efforts. And, nearly two-thirds (61.8 %) reported motivation to expand their scholarly work. However, fewer than one-half reported motivation to prepare a manuscript (49.6 %), and only one-quarter felt motivated to write a grant (27.9%).

**Table 2:** Conference attendance benefits as reported by conference presenters; N = 137^a^, 2014

To what extent did presenting at the AHPCCH …	Strongly agree/agree *N* (%)	Strongly disagree/disagree/neutral *N* (%)
Feedback/validation		
Provided me with valuable feedback on my work	97 (71.3)	39 (28.7)
Provided me with constructive feedback leading to improvements in my work	84 (61.8)	52 (38.2)
Provided me with encouragement to continue my work	115 (83.9)	22 (16.1)
Improved my confidence	88 (64.7)	48 (35.3)
Helped me to build my identity in this field	90 (65.7)	47 (34.3)
Validated my contribution to the field of criminal justice health	100 (73.0)	37 (27.0)
Led to opportunities for subsequent dissemination at a nationally-recognized peer-reviewed conference	55 (40.7)	80 (59.3)
Led to opportunities for subsequent dissemination in a peer-reviewed journal	53 (39.0)	83 (61.0)
Networking opportunities		
Provided me with networking opportunities with senior faculty	103 (75.7)	33 (24.3)
Provided me with networking opportunities with individuals with similar interests to mine	117 (86.0)	19 (14.0)
Provided me with networking opportunities with funders	31 (22.8)	105 (77.2)
Provided me with networking opportunities with journal editors and other publishers	31 (23.3)	102 (76.7)
Motivation		
Motivated me to write a grant	38 (27.9)	98 (72.1)
Motivated me to prepare a manuscript	68 (49.6)	69 (50.4)
Motivated me to expand my research and scholarly work	84 (61.8)	52 (38.2)
Provided me with new ideas for research and/or academic efforts in criminal justice health	108 (78.8)	29 (21.2)
Other tangible outcomes^b^		
Changed my clinical practice	18 (20.0)	72 (80.0)
Helped me to develop new clinical policies or guidelines in criminal justice health	22 (23.9)	70 (76.1)
Helped me to develop new or expanded curriculum or training opportunities in criminal justice health	35 (37.2)	59 (62.8)
Provided me with new ways of thinking about a problem I have faced in criminal justice health	72 (63.2)	42 (36.8)

^a^Study Sample: Some variables may not total to 137 because of sporadic missing data

^b^A number of respondents were self-rated as being ‘not applicable’ to answer these questions; for example, several questions pertained specifically to clinical practice

There were variable ranges of further dissemination of works presented at the conference (Table 3). Presenters reported means (SDs; medians) of 1.3 (1.9; 1.0) for publications; 1.7 (2.3; 1.0) follow-up presentations; 0.9 (1.4; 0.0) grants; and 1.1 (1.3; 1.0) new collaborations specifically related to their work presented at the AHPCCH. These descriptive statistics were re-computed removing those presenters who had ‘0’ scholarly outputs (Table 3). Among those who had at least 1 publication, the mean increased from 1.3 to 2.2 (range 1–15). Similarly, computed means for presentations, grants and new collaborations were 2.8, 2.0 and 1.9, respectively – all nearly doubled when reviewing outputs among those with 1 or more successes. For comparison purposes, Table 4 displays the percent distributions of these four outputs within frequency categories. For example, while the mean number of publications subsequent to the conference presentation was 2.2, the majority (51.9 %) of respondents published 1-3 manuscripts. Less than one in ten (7.0 %) of respondents reported 4+ publications subsequent to their AHPCCH presentation. There was a similar pattern for presentations, grants written, and new collaborations established.

**Table 3:** Scholarly outcomes of conference presenters; *N* = 137, 2014

Since presenting at the AHPCCH…	All Respondents *N *Mean (SD) Median Range	Respondents with at least 1 scholarly outcome^a^ *N *Mean (SD) Median Range
Publications specifically related to your work presented at the conference	129 1.3 (1.9) 1.0 0 – 15	70 2.2 (2.1) 2.0 1 – 15
Peer-reviewed presentations made in follow-up to the work presented at the conference	128 1.7 (2.3) 1.0 0 – 12	77 2.8 (2.3) 2.0 1 – 12
Grant written related to the work presented at the conference	127 0.9 (1.4) 0.0 0 – 10	57 2.0 (1.6) 2.0 1 – 10
New collaborations developed specifically related to the work presented at the conference	127 1.1 (1.3) 1.0 0 – 6	70 1.9 (1.2) 2.0 1 – 6

^a^Excluding those who responded ‘0’ to the specific outcome; i.e., univariate statistics represent only those who had at least 1 positive response to the individual outcome

**Table 4:** Scholarly outcomes of conference presenters; *N* = 137, 2014

Since presenting at the AHPCCH…	Number of scholarly outputs		
	0	1–3	4+
	*N* (%)	*N* (%)	*N* (%)
Publications specifically related to your work presented at the conference	53 (41.1)	67 (51.9)	9 (7.0)
Peer-reviewed presentations made in follow-up to the work presented at the conference	51 (39.8)	62 (48.4)	15 (11.7)
Grants written related to the work presented at the conference	70 (55.1)	53 (41.7)	4 (3.1)
New collaborations developed specifically related to the work presented at the conference	57 (44.9)	63 (49.6)	7 (5.5)

When asked to comment further about other scholarly outputs as a result of presenting at the conference, one respondent noted *“AHPCCH is the only venue I have found where I can gain access to the luminaries in the field and spend time really considering the best scientific approach to answering the difficult research questions I face in my every day work as a researcher in criminal justice healthcare. Thank you!”* Another presenter noted that the conference “*Helped [me] develop a mentorship committee for my NIDA career development award and meet potential co-authors/collaborators for future publications.*” A third respondent reported that presenting at the conference *“Increased visibility of my work within my own institution, which has led to increased and improved collaborations there”.* Finally, one presenter noted *“This conference has pushed me to grow as a researcher. Every year I leave feeling more inspired.”*

### Scholarship recipients

One-quarter (40; 28.6 %) of the survey respondents applied for and received a scholarship in order to attend at least one of the three conferences. Nearly all of these individuals (37 of 40; 92.5 %) were also presenters at the conference. They heard about the scholarship opportunity from faculty members with whom they work, mentors, peers, and the conference website. Scholarships seem to have been key for many of the recipients being able to attend the conference as over one-half of awardees (21 of 39; 53.8 %) would not have been able to attend the conference without scholarship funding. An additional one-third (13 of 39; 33.3 %) reported ‘maybe/depends’ on whether the scholarship confirmed their attendance. Nearly all (35 of 38; 92.1 %) recipients have continued to work in the field of criminal justice health, though fewer (17 of 39; 43.6 %) noted that receiving the scholarship impacted their recognition or visibility at their home institution. However, most recipients (33 of 38; 86.8 %) confirmed their current plans to contribute to the field of criminal justice health in the future. When queried further about their continued work, the responses were very powerful. One scholarship recipient noted *“I’m in the process of developing a non-profit organization aimed at promoting continuity of healthcare for correctional re-entry and bridging the gap between correctional healthcare and re-integrating into managed care upon release.”* Another recipient reported *“I’m working on several projects related to mortality as well as health information technology use among detained youths.”* Finally, a recipient noted *“I have several research projects underway pertaining to mental health and recidivism, mortality following release, and the effectiveness of a group intervention for prisoners with psychiatric disorders.”*

### Academic home institution climate toward criminal justice health

The final section of the survey queried all respondents about the climate in their home institutions toward the field of criminal justice health. Most reported that their choice to do work in criminal justice health was respected (75.4 %), with nearly two-thirds reporting collaborators with similar content interest and expertise (63.7 %) and opportunities to advance available to them as a result of their work in criminal justice health (60.0 %). However, most respondents reported receiving no institutional funding during periods when their own extramural funding is low (70.1 %) and more than one-half (58.6 %) were not part of a research core (i.e., 3 or more investigators) focused on criminal justice health (Table 5). Finally, only two of five (40.2 %) responding attendees reported not having a mentor at their home institution who supports their work in criminal justice health research.

**Table 5:** Home institutional criminal justice health climate assessment by AHPCCH conference attendees; *N* = 140^a^, 2014

At your academic home institution…^b^	A lot/A great deal *N* (%)	Not at all/Somewhat *N* (%)
Do you have collaborators with similar content interest and expertise?	77 (63.6)	44 (36.4)
Are opportunities to advance available to you as a result of the work you’re doing in criminal justice health?	72 (60.0)	48 (40.0)
Is your choice to do work in criminal justice health respected?	92 (75.4)	30 (24.6)
	Yes *N* (%)	No *N* (%)
Is mentorship available that supports your work in criminal justice health?	70 (59.8)	47 (40.2)
Are you part of a research core (i.e., 3 or more investigators) focused on criminal justice health?	48 (41.4)	68 (58.6)
Do/Did you currently receive institutional funding during periods when your own extramural funding is/was low?	29 (29.9)	68 (70.1)

^a^Study Sample: Some variables may not total to 140 because of sporadic missing data

^b^A number of respondents were self-rated as being ‘not applicable’ to answer these questions; for example, several conference attendees do not have an ‘academic home’

### Respondent reflections

At the end of the survey, respondents were asked to reflect on their own experiences and share with us thoughts on how the conference(s) impacted their work in criminal justice health. A formal theme analysis was not completed though their reflections were overwhelmingly positive (from 21 respondents).

*“Although the conference has not led directly to a publication, it was very helpful as a networking tool, and the discussion following my presentation resulted in some interesting ideas that I have implemented in my research.”*

*“Excellent conference. Very encouraging to see partnerships between corrections and academia. Research projects have been very difficult to accomplish in the past with correctional populations. The efforts displayed at the conference are very promising for the future.”*

*“The conference was an excellent opportunity for me to hear about relevant work being undertaken both in the USA and elsewhere. It was also a wonderful opportunity to meet potential supervisors and peers and discuss both their and my work.”*

*“There are many important intangible outcomes. I think that I am not alone when I say that the conference provides motivation and inspiration to continue this work--which is often much needed given little support in our academic home institutions.”*

## Discussion

A majority of conference presenters and scholars who completed the survey felt that the conference provided encouragement to continue their work, validated their identity as a contributor in the field of criminal justice health, provided constructive feedback, and improved their confidence. Moreover, the conference provided important networking opportunities and generated new ideas for research and/or academic efforts. Additional dissemination outputs catalyzed by presenting at the conference were mixed, with approximately half of the respondents noting publications, grants or other presentations following from the work presented at the conference. Most scholarship recipients reported continued professional commitment to the field of academic criminal justice health. These findings, while preliminary, are consistent with the positive attributes of scientific meetings published in the peer-reviewed literature (Wood and Morrison 2011; Scherer et al. 2007).

With regard to supports in their home institutions, it was noteworthy that a majority indicated respect for their academic pursuits and tangible support from peers with similar content expertise and mentorship opportunities. Just over half indicated a role in a criminal justice health research core. Despite these positive findings, a significant proportion of presenters and scholars did not have access to mentors and colleagues with similar interests and fully 70.1 % cited no institutional funding to support their work at times when there were gaps in external funding.

We hypothesize that, while a substantial proportion of presenters and scholars belong to high profile institutions with deep programs in criminal justice health research and scholarship, there are still many who look to this conference to fulfill important professional support functions available in most academic institutions such as mentoring and collaboration. In addition, with only a small proportion of clinical criminal justice health programs based in academic health science centers, only a limited number of clinicians are expanding the diversity of their roles into academic domains such as teaching and research. It is critical to continue to nurture and “reach” this group of individuals who may not have those resources available to them in their home institutions. This is particularly true of junior investigators and students who may have a passion for the field but lack the support to consider a career in the field. These needs can further be met through online education efforts, membership in consortia of criminal justice health scholars and certificates of added qualification following specific clinical training. For example, the American Osteopathic Association has developed accreditation for clinical criminal justice health fellowships in addition to certificates of added qualification (American Osteopathic Association 2012).

Among both the quantitative survey responses and the qualitative comments from conference attendees, respondents reported new research collaborations, refinements through constructive feedback, and a generation of new ideas for research and teaching arising as a result of conference attendance. While only a fifth of respondents noted changes to clinical practice or clinical policy, it is important to remember that the distribution of respondents in multiple disciplines, many unconnected with actual care or clinical policy development, may have diluted the impact on these conference outcomes.

There are several limitations of the study that deserve mention. First, the response rate of 56.0 % is lower than desirable; very limited information on non-respondents did not allow us to address the potential selection bias of respondents. Despite this, we did reach a representative group of individuals working in the field who attended the conference given the diverse roles and fields of the respondents. We chose to survey only the presenters and scholars who attended the conference, postulating that this group would be more likely to be committed to the advancement of scholarship in the field. We do not know if this assumption is accurate and have no information of the impact of the conference on participants who neither presented nor received a scholarship. We also have no way to verify whether self-reported conference presentations actually led directly to the subsequently reported dissemination outputs as reported to us by respondents. The multiple roles of presenters and scholars in their home institutions made it difficult to discriminate whether researchers versus presenters and scholars with other roles were more productive as a result of the conference. Likewise, we were unable to discern if those presenters or scholars who are dipping their toes in the water of academic pursuit were reinforced more positively than other respondents.

## Conclusion

In conclusion, our findings suggest that the Academic and Health Policy Conference on Correctional Health provides a valuable opportunity for researchers, policymakers and clinicians to network, share and improve upon their work, generate new ideas for research and validate criminal justice health as an academic field of study. In particular, for those individuals without a peer group or strategic support of their chosen work in home institutions, the conference serves as an academic home needed for successful professional development.

Going forward, we see the conference as providing an opportunity to initiate translational research, adapting important research findings to improve clinical management and clinical policies, given the many forces at play that may impede development of evidence-based practices.
